# Longitudinal patterns and predictors of response to standard-of-care therapy in lupus nephritis: data from the Accelerating Medicines Partnership Lupus Network

**DOI:** 10.1186/s13075-024-03275-z

**Published:** 2024-02-20

**Authors:** Peter M. Izmirly, Mimi Y. Kim, Philip M. Carlucci, Katherine Preisinger, Brooke Z. Cohen, Kristina Deonaraine, Devyn Zaminski, Maria Dall’Era, Kenneth Kalunian, Andrea Fava, H. Michael Belmont, Ming Wu, Chaim Putterman, Jennifer Anolik, Jennifer L. Barnas, Betty Diamond, Anne Davidson, David Wofsy, Diane Kamen, Judith A. James, Joel M. Guthridge, William Apruzzese, Deepak A. Rao, Michael H. Weisman, The Accelerating Medicines Partnership in RA/SLE Network, Darren Tabechian, Ralf Thiele, Jennifer Hossler, Brendan Boyce, Nida Meednu, Javier Rangel-Moreno, Christopher Ritchlin, Vivian Bykerk, Laura Donlin, Susan Goodman, Lionel Ivashkiv, Alessandra Pernis, Ed DiCarlo, Dana Orange, John Carrino, Oganna Nwawka, Endo Yoshimi, Rahul Satija, Lionel Ivashkiv, Robert Darnell, Mark Figgie, Michael McNamara, Larry W. Moreland, Mandy J. McGeachy, Jay Kolls, Aaron Wise, Andrew Cordle, Peter Gregersen, Diane Horowitz, Andrew D. Filer, Jason Turner, Holly Adams, Costantino Pitzalis, Stephen Kelly, Rebecca Hands, Michael Brenner, Derrick Todd, Kevin Wei, Deepak Rao, Fumitaka Mizoguchi, V. Michael Holers, Kevin D. Deane, Jennifer A. Seifert, Nirmal K. Banda, Gary S. Firestein, David Boyle, Ami Ben-Artzi, Lindsy Forbess, Ellen Gravallese, Karen Salomon-Escoto, Harris Perlman, Arthur Mandelin, Emily Bacalao, Deborah Parks, John Atkinson, Joan Bathon, Eric Matteson, Louis Bridges, Laura B. Hughes, David Fox, Robert Ike, Chun-Hao Lee, Derek Fine, Manny Monroy-Trujillo, Jennifer Anolik, Ummara Shah, Michael Weisman, Mariko Ishimori, Jill P. Buyon, Robert M. Clancy, Peter Izmirly, Michael Belmont, Nicole Bornkamp, Evan Der, Beatrice Goilav, Nicole Jordan, Daniel Schwartz, James Pullman, Dawn Smilek, Patti Tosta, Matthias Kretzler, Celine C. Berthier, F. Steve Woodle, Dave Hildeman, Michael Brenner, Deepak Rao, William Robinson, Garry Nolan, Veronica Gonzales, Michael Brenner, Deepak Rao, Kevin Wei, Jim Lederer, Joshua Keegan, Adam Chicoine, Yanyan Liu, Gerald Watts, Nir Hacohen, Arnon Arazi, David Lieb, Thomas Eisenhaure, Thomas Tuschl, P. J. Utz, Mina Rohani-Pichavant, Rohit Gupta, Holden Maecker, Maria Sargent, Soumya Raychaudhuri, Yvonne Lee, Kamil Slowikowski, Chamith Fonseka, Fan Zhang, Maria Guitierrez-Arcelus, Justine Buschman, Jennifer Chi, Su-Yau Mao, Susana Serrate-Sztein, Yan Wang, Quan Chen, John Peyman, Ellen Goldmuntz, Patrick Dunn, Michelle Petri, Jill Buyon, Richard Furie

**Affiliations:** 1 New York University Grossman School of Medicine, 550 First Avenue, MSB 593D, New York, NY 10016 USA; 2 Albert Einstein College of Medicine, Bronx, New York, NY USA; 3 University of California San Francisco, San Francisco, CA USA; 4 University of California San Diego, San Diego, CA USA; 5 Johns Hopkins University, Baltimore, MD USA; 6 Azrieli Faculty of Medicine, Zefat, Israel; 7 University of Rochester Medical Center, Rochester, NY USA; 8 Zucker School of Medicine at Hofstra/Northwell, Manhasset, NY USA; 9 Medical University of South Carolina, Charleston, SC USA; 10 Oklahoma Medical Research Foundation, Oklahoma City, OK USA; 11 Pfizer Inc., New York, NY USA; 12 Brigham and Women’s Hospital, Boston, MA USA; 13 Stanford University, Palo Alto, CA USA

**Keywords:** Lupus nephritis, Systemic lupus erythematosus (SLE), Outcome, Renal biopsy

## Abstract

**Background:**

Leveraging the Accelerating Medicines Partnership (AMP) Lupus Nephritis (LN) dataset, we evaluated longitudinal patterns, rates, and predictors of response to standard-of-care therapy in patients with lupus nephritis.

**Methods:**

Patients from US academic medical centers with class III, IV, and/or V LN and a baseline urine protein/creatinine (UPCR) ratio ≥ 1.0 (*n* = 180) were eligible for this analysis. Complete response (CR) required the following: (1) UPCR < 0.5; (2) normal serum creatinine (≤ 1.3 mg/dL) or, if abnormal, ≤ 125% of baseline; and (3) prednisone ≤ 10 mg/day. Partial response (PR) required the following: (1) > 50% reduction in UPCR; (2) normal serum creatinine or, if abnormal, ≤ 125% of baseline; and (3) prednisone dose ≤ 15 mg/day.

**Results:**

Response rates to the standard of care at week 52 were CR = 22.2%; PR = 21.7%; non-responder (NR) = 41.7%, and not determined (ND) = 14.4%. Only 8/180 (4.4%) patients had a week 12 CR sustained through week 52. Eighteen (10%) patients attained a week 12 PR or CR and sustained their responses through week 52 and 47 (26.1%) patients achieved sustained PR or CR at weeks 26 and 52. Week 52 CR or PR attainment was associated with baseline UPCR > 3 (OR_adj_ = 3.71 [95%CI = 1.34–10.24]; *p* = 0.012), > 25% decrease in UPCR from baseline to week 12 (OR_adj_ = 2.61 [95%CI = 1.07–6.41]; *p* = 0.036), lower chronicity index (OR_adj =_ 1.33 per unit decrease [95%CI = 1.10–1.62]; *p* = 0.003), and positive anti-dsDNA antibody (OR_adj_ = 2.61 [95%CI = 0.93–7.33]; *p* = 0.069).

**Conclusions:**

CR and PR rates at week 52 were consistent with the standard-of-care response rates observed in prospective registrational LN trials. Low sustained response rates underscore the need for more efficacious therapies and highlight how critically important it is to understand the molecular pathways associated with response and non-response.

**Supplementary Information:**

The online version contains supplementary material available at 10.1186/s13075-024-03275-z.

## Background

The Accelerating Medicines Partnership (AMP) RA/SLE Network was established with the goal of applying new technologies, such as single-cell RNA sequencing of diseased kidney tissue, to improve diagnostic and therapeutic tools that would ultimately enhance lupus nephritis (LN) outcomes [1]. The AMP LN cohort, initiated in the United States (US) through the multi-center enrollment of patients with LN undergoing standard-of-care kidney biopsies, reflects real-world management and outcomes of a diverse population. In a prior publication, Deonaraine et al. provided reassurances regarding the safety of obtaining kidney tissue for AMP research during clinically indicated biopsies [2]. In another analysis of the AMP dataset, Carlucci et al. noted a high frequency of proliferative as well as membranous nephritis in enrolled AMP patients with baseline levels of proteinuria lower (urine protein/creatinine ratios between 0.5 and 1) than the typical threshold required for inclusion in registrational LN clinical trials [3].

In this interrogation of the AMP dataset, we determined the percentages of patients who attained pre-specified definitions of partial or complete responses at specific visits over 1 year of treatment follow-up and examined the longitudinal patterns of response. In addition, clinical and laboratory characteristics associated with clinical responses were identified. In contrast to global LN clinical trials, the AMP LN cohort affords an opportunity to generate outcome data representative of a US multicenter, multi-racial, multi-ethnic real-world experience.

## Methods

### Patient population

Patients with LN undergoing kidney biopsies as part of the standard of care were eligible to enroll in the prospective AMP LN study. The decision to biopsy was at the discretion of the treating rheumatologist or nephrologist to confirm suspected lupus nephritis de novo, an activity not responding to treatment, or relapse of disease. Inclusion in AMP required the following: (1) age ≥ 18; (2) fulfillment of the revised American College of Rheumatology [4, 5] or the Systemic Lupus Erythematosus International Cooperating Clinics [6] classification criteria for SLE; (3) a urine protein/creatinine ratio (UPCR) > 0.5 at the time of biopsy. For the analyses reported herein, the classification of responder status was restricted to patients with baseline random or 24-h UPCR ≥ 1.0 since for patients with ratios between 0.5 and 0.999, proteinuric response has not been defined. Only patients with renal biopsies that demonstrated the International Society of Nephrology/Renal Pathology Society (ISN/RPS) classes III, IV, V, or combined III or IV with V read by the pathologist at each participating site were considered in this analysis [7, 8]. Exclusion criteria included the following: (1) a history of kidney transplant, (2) rituximab treatment within 6 months of biopsy, (3) pregnancy at the time of biopsy. The study protocol was approved by the institutional review boards and ethics committees of participating sites in adherence with the Declaration of Helsinki.

Baseline demographics from a predetermined set of categories, including self-reported race (Asian, Black, White, Other)/ethnicity (Hispanic, non-Hispanic) as required for NIH-funded studies, and clinical characteristics were recorded at the time of biopsy. Laboratory tests and medications were documented at each visit (baseline, week 12, week 26, and week 52) and were performed at the participating sites. Given medication changes occurred after the baseline visit in response to receipt of the kidney biopsy results, we chose the week 12 treatment to represent the induction regimen. For steroids, the higher dose at either baseline or week 12 was considered the induction dose for similar reasons. Pulse steroids were also captured separately.

### Outcomes

Complete response (CR) required the following: (1) UPCR < 0.5; (2) normal creatinine (≤ 1.3 mg/dL) or, if abnormal, ≤ 125% of baseline; and (3) prednisone ≤ 10 mg/day at the time of the study visit. Partial response required the following: (1) > 50% reduction in UPCR; (2) normal creatinine (≤ 1.3 mg/dL) or, if abnormal, ≤ 125% of baseline; and (3) prednisone dose ≤ 15 mg/day at the time of the study visit. Patients who did not achieve a CR or PR at the specific timepoints were considered non-responders (NR) or not determined (ND) if data were missing. These response definitions were based on the ACCESS Trial [9]. In agreement with the ACCESS trial, we specifically decided not to include the microscopic review of the urine sediment given the absence of uniformity across sites in assessing urinary sediment and the challenge of attribution especially in a population of young women. The prednisone threshold for CR at ≤ 10 mg prednisone was also based on the ACCESS trial. However, the ≤ 15 mg prednisone maximum for defining PR was agreed upon unanimously by the site investigators.

Although proteinuria was measured by either a UPCR on spot urine or a timed urine collection, consistency of the method across the study for an individual was required. While determination from a timed urine collection was preferred, if this method was not performed at all time points for an individual participant, calculations from spot urine were utilized.

### Statistical analysis

Descriptive statistics are presented as mean and standard deviation or median and interquartile range for continuous variables and frequencies for categorical variables. Pairwise agreement between response status at different time points was estimated by computing the kappa statistic. Logistic regression was performed to identify variables that independently discriminated persistent responders and never responders and estimate adjusted odds ratios (OR_adj_). Given the small number of patients who had a CR or PR at all three follow-up visits, persistent responders were defined as those patients who achieved CR or PR at both 26 and 52 weeks; never responders were patients who did not achieve either CR or PR at any visit. In addition, logistic and multinomial logistic regression models were fit to the data to identify independent predictors of response status at 52 weeks only. Variable selection during model development was based on both statistical and clinical considerations, but the final model included only those variables that remained significant at the *p* < 0.10 level (a more liberal threshold for retaining variables in the final model was applied given the limited number of events). In addition to those variables listed in Table 1, potential predictors included baseline creatinine (> 1.3 vs ≤ 1.3), protein decreasing by 25% at 12 weeks, membranous vs proliferative and class III + V/IV + V biopsies, and induction prednisone dose (≥ 30 mg, < 30 and > 10 mg, and ≤ 10 mg). Missing data in the logistic regression analysis was handled using list-wise deletion. Sensitivity analysis was also performed based on non-responder imputation and multiple imputation (MI) with 40 imputed data sets. The MI model included the outcome variable, predictors from all logistic regression models, and several additional auxiliary variables (prednisone use, activity index, creatine level). All analyses were performed in SAS, version 9.4.

**Table 1:** Demographics and baseline characteristics of patients with baseline UPCR ≥ 1

**Demographics (*n* = 180)**	
**Sex: female**	156 (86.7%)
**Age, mean (SD)**	35.2 (11.4)
**Ethnicity: Hispanic**	59 (32.7%)
**Race**	
Asian	29 (16.1%)
Black	76 (42.2%)
White	53 (29.4%)
Other/unknown	22 (12.2%)
**First biopsy**	62 (34.0%)
**UPCR, mean [IQR]**	3.5 [1.60–4.38]
**Nephrotic proteinuria**	82 (45.6%)
**Serum creatinine mg/dL, mean [range] (*n* = 179)**	1.25 [0.4–7.4]
**High serum creatinine (*n* = 179)**^**a**^	46 (25.7%)
**Low C3 (*n* = 178)**^**a**^	116 (65.2%)
**Low C4 (*n* = 178)**^**a**^	102 (57.3%)
**Serum albumin g/dL, mean [range] (*N* = 171)**	3.1 [1.0–4.7]
**Positive anti-dsDNA (*n* = 176)**	124 (70.5%)
**Biopsy class**	
[III]	30 (16.7%)
[IV]	35 (19.4%)
[V]	51 (28.3%)
[III][IV]	3 (1.7%)
[III][V]	36 (20.0%)
[IV][V]	25 (13.9%)
Activity Index, mean [range] (*n* = 143)	5.4 [0–18]
Chronicity Index, mean [range] (*n* = 143)	3.3 [0–10]
Extra renal activity on hybrid SELENA-SLEDAI^**b**^	87 (48.3%)
**Medications**^**c**^	
Hydroxychloroquine Daily average dose [range]	137 (76.1%) 356.1 mg [85.7–800]
Prednisone/methylprednisolone Daily average dose [range] Pulse steroids	135 (75.0%) 24.4 mg [2.5–120] 21 (11.7%)
Mycophenolate mofetil Daily average dose [range] Mycophenolic acid Daily average dose [range]	116 (6.44%) 2435.3 mg [500–3000] 8 (4.4%) 1215 mg [360–2880]
Cyclophosphamide	24 (13.3%)
Azathioprine	6 (3.3%)
Tacrolimus	19 (10.6%)
Belimumab	4 (2.2%)
Leflunomide	1 (0.6%)

Unless otherwise indicated, variables had data available for all 180 patients

*UPCR* urine protein/creatinine ratio, *anti-dsDNA* anti-double-stranded DNA autoantibodies, *SELENA-SLEDAI* Safety of Estrogens in Lupus Erythematosus: National Assessment- Systemic Lupus Erythematosus Disease Activity Index

^a^Classified by local laboratory cutoff

^b^Includes all hybrid SELENA-SLEDAI domains that include clinical activity excluding serologic and renal urine activity

^c^Captured at week 12 visits, for steroids the higher dose at two visits (baseline and week 12) was considered induction dosing given patients who had their doses increased after the biopsy would not be captured at the baseline visit

## Results

### Baseline characteristics

**Fig. 1:**
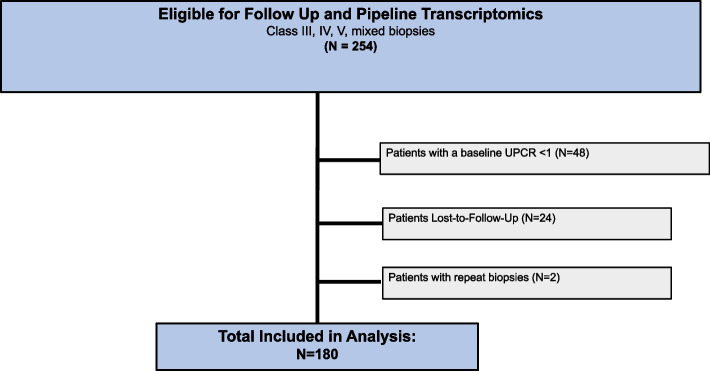
Flow diagram of study enrollment

One hundred eighty patients met the inclusion criteria (Fig. 1). Of these, 86.7% were women, 29.4% were White, and 32.7% were Hispanic (Table 1). The mean age was 35.2 (SD 11.4) years. Using administered medications at week 12 to capture induction therapy, the majority (64%) were treated with mycophenolate mofetil, and 13% with cyclophosphamide. Seventy-five percent of the cohort received steroids with an average dose of prednisone equivalent of 24.4 mg, and 76% were taking hydroxychloroquine. Biopsy classes were as follows: III = 16.7%, IV = 19.4%, V = 28.3%, and III + V/IV + V = 33.9%. Sixty-six percent of patients had a previous biopsy. Average baseline creatinine was 1.25. A positive anti-dsDNA antibody (measured locally) was present in 70.5%, 65.2% had a low C3 level, and 57.3% had a low C4 level. The average baseline UPCR was 3.5. Overall, 48.3% of the 180 patients had extra renal activity on the hybrid SELENA- SLEDAI at baseline.

### Longitudinal patterns of response

**Fig. 2:**
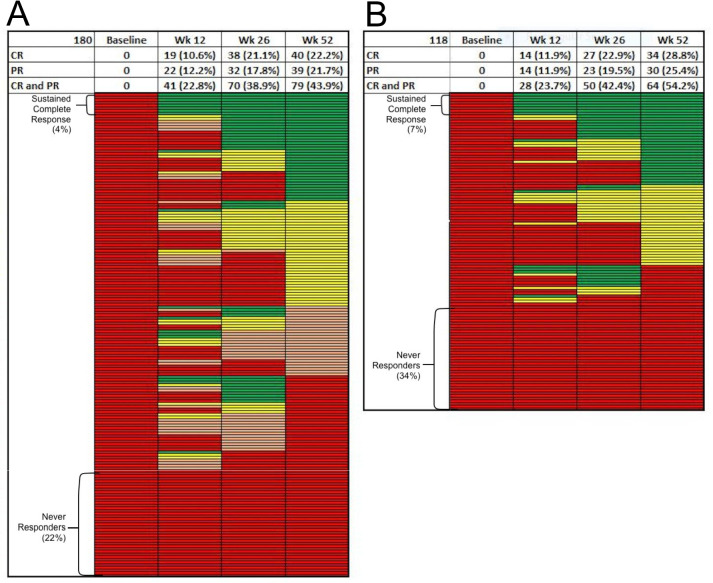
Response rates and graphical heat map displays of responses at each visit

The response rates and graphical heat map displays of responses at each visit are shown in Fig. 2A. Response rates at week 52 were as follows: CR = 22.2%; PR = 21.7%; NR = 41.7%; and ND = 14.4%. Only 8/180 (4.4%) of patients had a confirmed week 12 CR response sustained through week 52. Eighteen (10%) patients attained a PR or CR at week 12 and sustained their responses through week 52, and 47 (26.1%) patients achieved a PR or CR at week 26, which was sustained at week 52. Overall, 40/180 (22.2%) were confirmed NR at all time points, which increased to 67 (37.2%) when non-responder imputation (NRI) was applied for missing data (Supplemental Fig. 1A). Figure 2B is a display restricted to patients (*n* = 118) for whom responder status was available at all time points. Although not used in further analysis of renal responder status, applying less stringent definitions of proteinuric responses, independent of creatinine or prednisone dose at 52 weeks, 69/180 (38.3%) had a UPCR ≤ 0.8 and 62/180 (34.4%) had a UPCR ≤ 0.7 compared to 48/180 (26.7%) attaining a UPCR of ≤ 0.5. The most common reason for regressing at 52 weeks from an initial CR/PR was the return of proteinuria above the response definition.

Based on the observed data, there was a fair agreement between response status at weeks 12 and 26 (kappa = 0.41 [95% CI 0.27–0.56]) and between weeks 26 and 52 (kappa = 0.36 [95% CI = 0.21–0.51]). As expected, agreement in response status between weeks 12 and 52 was weaker (kappa = 0.16 [95% CI = 0.015–0.30]) (Table 2). When NRI was used to handle missing data, agreement in response status across visits was similar or slightly lower (Supplementary Table 1).

**Table 2:** Agreement in response status (complete or partial) across visits

	% Response at both visits	% Non-response at both visits	% Discordant response status	Kappa (95% CI) for agreement in response status across visits
Week 12 and week 26	20.8%	51.5%	27.7%	0.41 (0.27, 0.56)
Week 12 and week 52	16.5%	40.9%	42.5%	0.16 (0.015, 0.30)
Week 26 and week 52	33.8%	33.8%	32.4%	0.36 (0.21, 0.51)

Assuming not determined = missing

### Patient characteristics associated with persistent responses at weeks 26 and 52

As shown in Table 3, logistic regression analysis indicated that the following patient characteristics independently favored CR or PR responses at both weeks 26 and 52 (persistent responders) compared to NR at all time points: a > 25% decrease in UPCR between baseline and week 12 (OR_adj_ = 7.37 [95% CI = 2.31–23.49]; *p* < 0.001), positive anti-dsDNA antibody (OR_adj_ = 4.70 [95% CI = 1.19–18.51]; *p* = 0.027), first biopsy (OR_adj_ = 3.12 [95% CI = 0.89–10.89]; *p* = 0.075) and no use of cyclophosphamide for induction (OR_adj_ = 5.08 [95% CI = 0.80–32.26]; *p* = 0.084). Estimated odds ratios observed in sensitivity analyses in which missing data were addressed with non-response imputation and multiple imputation showed similar results (Supplementary Table 2).

**Table 3:** Predictors of response (complete or partial) at both weeks 26 and 52 versus no response at all visits from logistic regression

Predictor variable	Odds ratio estimate (95% confidence interval)	*P* value
First biopsy	3.12 (0.89–10.89)	0.075
Anti-dsDNA antibody positive	4.70 (1.19–18.51)	0.027
No Cyclophosphamide induction	5.08 (0.80–32.26)	0.084
UPCR > 25% decrease from baseline to week 12	7.37 (2.31–23.49)	< 0.001

Based on available data for responder adjudication and all covariates

*UPCR* urine protein/creatine ratio, *anti-dsDNA antibody* anti-double-stranded DNA autoantibody

### Patient characteristics associated with response at week 52

Patient characteristics favoring CR or PR responses compared to NR at week 52 from logistic regression analysis were as follows: UPCR > 3 at baseline (OR_adj_ = 3.71 [95%CI = 1.3–10.24]; *p* = 0.012), > 25% decrease in UPCR from baseline to week 12 (OR_adj_ = 2.61 [95%CI = 1.07–6.41]; *p* = 0.036), lower chronicity index (OR_adj_ = 1.33 per unit decrease [95%CI = 1.10–1.62]; *p* = 0.003), and a positive anti-dsDNA antibody (OR_adj_ = 2.61 [95%CI = 0.93–7.33]; *p* = 0.069) (Table 4). Sensitivity analyses using methods to address missing data again showed similar trends, but the estimated odds ratio of UPCR > 3 was lower with multiple imputation (Supplementary Table 3). Limiting these analyses to Class V only, estimated odds ratios of predictor variables were larger but less statistically significant because of the smaller sample size (Supplementary Table 4).

**Table 4:** Predictors of week 52 response (complete or partial) versus no response from logistic regression analysis

Predictor variable	Odds ratio estimate (95% confidence interval)	*P* value
Anti-dsDNA antibody positive	2.61 (0.93–7.33)	0.069
UPCR > 25% decrease from baseline to week 12	2.61 (1.07–6.41)	0.036
Chronicity Index per unit decrease	1.33 (1.10–1.62)	0.003
UPCR > 3 at baseline	3.71 (1.34–10.24)	0.012

Based on available data for responder adjudication and all covariates

*UPCR* urine protein/creatinine ratio, *anti-dsDNA antibody* anti-double-stranded DNA autoantibody

In exploratory analyses, multinomial logistic regression with week 52 response status considered as three separate categories—CR, PR, and NR (in contrast to combining CR and PR)—suggested baseline positive anti-dsDNA antibody, > 25% decrease in UPCR from baseline to week 12, and chronicity index discriminated CR versus NR, while UPCR > 3 at baseline discriminated PR versus NR (Supplementary Table 5).

## Discussion

The AMP LN cohort provided outcome data representative of a large US multicenter, multi-racial, multi-ethnic real-world experience. In 180 patients, the response rates at week 52 were similar to those observed in pivotal FDA trials with complete response in only a fifth of the cohort and nearly half non-responders. Very few patients had a week 12 CR response sustained through the entire year of the study, and only 26% attained a PR or CR at both week 26 and week 52. Agreement in response status between 12 and 52 weeks was low. A > 25% decrease in UPCR from baseline to week 12 and/or a baseline positive anti-dsDNA antibody predicted both persistent CR or PR responses at weeks 26 and 52 and a CR or PR at 52 weeks only. First biopsy and/or no use of cyclophosphamide induction was only associated with sustained responses at weeks 26 and 52, whereas a baseline UPCR > 3 and lower chronicity index were only associated with CR or PR responses at 52 weeks.

In BLISS-LN [10], a phase III 2-year study of belimumab in patients with proliferative and/or membranous nephritis, the probabilities of achievement of the primary endpoint (Primary Efficacy Renal Response) as well as secondary endpoint (Complete Renal Response) were determined. While entry criteria, endpoints, and treatment interventions differed from the AMP study, achievement in BLISS-LN of sustained CRR, which most closely approximates the AMP endpoint, was approximately 13% at 1 year in the placebo group.

The CR rate (22.2%) in AMP was very similar to those reported in LN clinical trials despite the differences in definitions across studies. In recently published clinical trials of belimumab, voclosporin, and obinutuzumab, CR rates of 20% (week 104), 23% (week 52), and 23% (week 52) in the placebo/standard of care arms, respectively, were observed [10–12]. PR rates of 17% (week 104), 50% (week 52), and 13% (week 52) in the placebo/standard of care arms were observed in the belimumab, voclosporin, and obinutuzumab studies, respectively, compared with 21.7% in AMP [10–12].

There are several limitations that could have influenced the results of this study. Doses of medications were recorded only at the respective visits, and thus there was likely an underestimation of the highest dose of administered steroids. Furthermore, potential changes in immunosuppression between visits such as intravenous regimens may not have been captured. As a result, changes in medications between visits, particularly after 26 weeks when a patient could have been considered an induction responder, were not analyzed in predictors of responses. The upper limit of normal for creatinine in some laboratories may be lower than 1.3 mg/dL, and it is acknowledged that given the high frequency of young adult females, the level chosen may be abnormal in this population. Applying a lower normal value would have resulted in even lower response outcomes. The small number of patients achieving a sustained CR or sustained PR precluded analyses of predictors of persistent response. Although of interest, there were too few patients to analyze those that initially responded but lost response at 52 weeks. Missing data is also a limitation although this was addressed using methods as previously described [13]. The negative association of cyclophosphamide with renal response may have been due to confounding by indication, especially in a cohort where the majority of patients had a prior history of LN. Complete response with proteinuria < 0.5 was a predefined outcome at the start of this study which began in 2014 to be consistent with current clinical trials at that time and will be used for future AMP biomarker studies [14]. Since then, there has been emerging evidence that proteinuria < 0.8 at 12 months is predictive of favorable long-term renal outcomes [15–17]. In this study, even liberalizing the definition of response to < 0.8 independent of creatinine or prednisone dose still resulted in a poor response rate at 38%.

The strengths of this study are that data were generated from academic institutions with familiarity in the treatment of lupus nephritis. This study represents real-world standard of care and includes sicker patients who otherwise would be excluded from clinical trials. In addition, the AMP cohort comprised a diverse racial and ethnic group of patients. This study also evaluated sustained response [18] as well as predictors of response, items which have not been commonly evaluated in LN trials.

## Conclusions

In summary, clinical data from the AMP Lupus Network revealed rates of 52-week CR, PR, and CR and PR that were consistent with standard of care/placebo response rates from recently conducted LN trials. Low sustained CR rates not only underscore the need for more efficacious therapies but highlight how critically important it is to understand the molecular pathways that are associated with response and non-response.

### Supplementary Information

**Additional file 1: Supplemental Table 1.** Agreement in response status (complete or partial) across visits using non-responder imputation for missing data. **Supplemental Table 2.** Predictors of response (complete or partial) at both weeks 26 and 52 versus no response at all visits from logistic regression using non-responder imputation for missing response data and multiple imputation for missing covariate data. **Supplemental Table 3.** Predictors of response (complete or partial) at week 52 versus no response from logistic regression analysis using non-responder imputation for missing response data and multiple imputation for missing covariate data. **Supplemental Table 4.** Predictors of response (complete or partial) at week 52 versus no response from logistic regression analysis for Class V cases only. **Supplemental Table 5.** Predictors of week 52 response using multinomial regression with available data. **Supplemental Figure 1.** Temporal patterns in the response status of patients with systemic lupus erythematosus receiving standard of care therapy employing nonresponder imputation for missing data for 180 patients included. Green indicates complete response, yellow indicates partial response and red indicates no response.

## Data Availability

The NIH is in the process of releasing the clinical datasets analyzed during the current study.

## Abbreviations

AMP: Accelerating Medicines Partnership
CR: Complete response
dsDNA: Double-stranded DNA
LN: Lupus nephritis
NR: Non-responder
ND: Not determined
PR: Partial response
SELENA-SLEDAI: Safety of Estrogens in Lupus Erythematosus: National Assessment-Systemic Lupus Erythematosus Disease Activity Index
UPCR: Urine protein to creatinine ratio

## References

[1] Hoover P, Der E, Berthier CC, Arazi A, Lederer JA, James JA, Buyon J, Petri M, Belmont HM, Izmirly P (2020). Accelerating Medicines Partnership: organizational structure and preliminary data from the phase 1 studies of lupus nephritis. Arthritis Care Res (Hoboken).

[2] Deonaraine KK, Carlucci PM, Fava A, Li J, Wofsy D, James JA, Putterman C, Diamond B, Davidson A, Fine DM, et al. Safety of procuring research tissue during a clinically indicated kidney biopsy from patients with lupus: data from the Accelerating Medicines Partnership RA/SLE Network. Lupus Sci Med. 2021;8(1):e000522. 10.1136/lupus-2021-000522 PMC8354250 34389634

[3] Carlucci P, Li J, Fava A, Deonaraine K, Wofsy D, James J, Putterman C, Diamond B, Davidson A, Fine DM et al: High incidence of proliferative and membranous nephritis in SLE patients with low proteinuria in the Accelerating Medicines Partnership. Rheumatology (Oxford) 2022, in press. 10.1093/rheumatology/keac067 PMC9629353 35212719

[4] Hochberg MC (1997). Updating the American College of Rheumatology revised criteria for the classification of systemic lupus erythematosus. Arthritis Rheum.

[5] Tan EM, Cohen AS, Fries JF, Masi AT, McShane DJ, Rothfield NF, Schaller JG, Talal N, Winchester RJ (1982). The 1982 revised criteria for the classification of systemic lupus erythematosus. Arthritis Rheum.

[6] Petri M, Orbai A-M, Alarcón GS, Gordon C, Merrill JT, Fortin PR, Bruce IN, Isenberg D, Wallace DJ, Nived O (2012). Derivation and validation of the Systemic Lupus International Collaborating Clinics classification criteria for systemic lupus erythematosus. Arthritis Rheum.

[7] Weening JJ, D'Agati VD, Schwartz MM, et al. The classification of glomerulonephritis in systemic lupus erythematosus revisited. Kidney international. 2004;65(2):521-30. 10.1111/j.1523-1755.2004.00443.x. 10.1111/j.1523-1755.2004.00443.x 14717922

[8] Bajema IM, Wilhelmus S, Alpers CE, et al. Revision of the International Society of Nephrology/Renal Pathology Society classification for lupus nephritis: clarification of definitions, and modified National Institutes of Health activity and chronicity indices. Kidney international. 2018;93(4):789-96. 10.1016/j.kint.2017.11.023. 10.1016/j.kint.2017.11.023 29459092

[9] Access Trial Group (2014). Treatment of lupus nephritis with abatacept: the Abatacept and Cyclophosphamide Combination Efficacy and Safety Study. Arthritis Rheumatol.

[10] Furie R, Rovin BH, Houssiau F, Malvar A, Teng YKO, Contreras G, Amoura Z, Yu X, Mok C-C, Santiago MB (2020). Two-year, randomized, controlled trial of belimumab in lupus nephritis. N Engl J Med.

[11] Rovin BH, Teng YKO, Ginzler EM, Arriens C, Caster DJ, Romero-Diaz J, Gibson K, Kaplan J, Lisk L, Navarra S (2021). Efficacy and safety of voclosporin versus placebo for lupus nephritis (AURORA 1): a double-blind, randomised, multicentre, placebo-controlled, phase 3 trial. Lancet.

[12] Furie RA, Aroca G, Cascino MD, Garg JP, Rovin BH, Alvarez A, Fragoso-Loyo H, Zuta-Santillan E, Schindler T, Brunetta P (2022). B-cell depletion with obinutuzumab for the treatment of proliferative lupus nephritis: a randomised, double-blind, placebo-controlled trial. Ann Rheum Dis.

[13] Kim M, Merrill JT, Wang C, Viswanathan S, Kalunian K, Hanrahan L, Izmirly P (2019). SLE clinical trials: impact of missing data on estimating treatment effects. Lupus Sci Med.

[14] Fava A, Rao DA, Mohan C, Zhang T, Rosenberg A, Fenaroli P, Belmont HM, Izmirly P, Clancy R, Trujillo JM (2022). Urine proteomics and renal single-cell transcriptomics implicate interleukin-16 in lupus nephritis. Arthritis Rheumatol.

[15] Ugolini-Lopes MR, Seguro LPC, Castro MXF, Daffre D, Lopes AC, Borba EF, Bonfa E (2017). Early proteinuria response: a valid real-life situation predictor of long-term lupus renal outcome in an ethnically diverse group with severe biopsy-proven nephritis?. Lupus Sci Med.

[16] Tamirou F, Lauwerys BR, Dall'Era M, Mackay M, Rovin B, Cervera R, Houssiau FA, Investigators MNT (2015). A proteinuria cut-off level of 0.7 g/day after 12 months of treatment best predicts long-term renal outcome in lupus nephritis: data from the MAINTAIN Nephritis Trial. Lupus Sci Med.

[17] Dall'Era M, Cisternas MG, Smilek DE, Straub L, Houssiau FA, Cervera R, Rovin BH, Mackay M (2015). Predictors of long-term renal outcome in lupus nephritis trials: lessons learned from the Euro-Lupus Nephritis cohort. Arthritis Rheumatol.

[18] Kim M, Merrill J, Kalunian K, Hahn B, Roach A, Izmirly P (2017). Lupus Foundation of America Collective Data Analysis Initiative G: Brief report: Longitudinal patterns of response to standard of care therapy for systemic lupus erythematosus: implications for clinical trial design. Arthritis Rheumatol.

