# Supplementary material for: Longitudinal patterns and predictors of response to standard-of-care therapy in lupus nephritis: data from the Accelerating Medicines Partnership Lupus Network
Source: Arthritis Res Ther. 2024 Feb 20;26:54. doi: 10.1186/s13075-024-03275-z (PMC10877793; doi:10.1186/s13075-024-03275-z)
Supplement: Supplementary file 1 — Additional file 1: Supplemental Table 1. Agreement in response status (complete or partial) across visits using non-responder imputation for missing data. Supplemental Table 2. Predictors of response (complete or partial) at both weeks 26 and 52 versus no response at all visits from logistic regression using non-responder imputation for missing response data and multiple imputation for missing covariate data. Supplemental Table 3. Predictors of response (complete or partial) at week 52 versus no response from logistic regression analysis using non-responder imputation for missing response data and multiple imputation for missing covariate data. Supplemental Table 4. Predictors of response (complete or partial) at week 52 versus no response from logistic regression analysis for Class V cases only. Supplemental Table 5. Predictors of week 52 response using multinomial regression with available data. Supplemental Figure 1. Temporal patterns in the response status of patients with systemic lupus erythematosus receiving standard of care therapy employing nonresponder imputation for missing data for 180 patients included. Green indicates complete response, yellow indicates partial response and red indicates no response. [file 13075_2024_3275_MOESM1_ESM.docx]

**Supplemental Table 1: Agreement in response status (complete or partial) across visits using non-responder imputation for missing data**

|  | % Response at both visits | % Non-Response at both visits | % Discordant Response status | Kappa (95% CI) for agreement in response status across visits |
| --- | --- | --- | --- | --- |
| Week 12 and Week 26 | 15.0% | 53.3% | 31.7% | 0.28 (0.14, 0.42) |
| Week 12 and Week 52 | 11.7% | 45.0% | 43.3% | 0.072 (-0.06, 0.20) |
| Week 26 and Week 52 | 26.1% | 43.3% | 30.6% | 0.37 (0.24, 0.51) |

**Supplemental Table 2: Predictors of response (complete or partial) at both weeks 26 and 52 versus no response at all visits from logistic regression using non-responder imputation for missing response data and multiple imputation for missing covariate data**

| Predictor Variable | Odds Ratio Estimate  (95% Confidence interval) | P value |
| --- | --- | --- |
| First biopsy | 2.02 (0.72- 5.56) | 0.17 |
| Anti-dsDNA antibody positive | 4.01 (1.33-12.13) | 0.01 |
| No Cyclophosphamide induction | 3.28 (0.75-14) | 0.12 |
| UPCR > 25% decrease from  baseline to week 12 | 8.84 (3.06-25.52) | <0.001 |

UPCR=urine protein/creatinine ratio

**Supplemental Table 3: Predictors of response (complete or partial) at week 52 versus no response from logistic regression analysis using non-responder imputation for missing response data and multiple imputation for missing covariate data**

| Predictor Variable | Odds Ratio Estimate  (95% Confidence interval) | P value |
| --- | --- | --- |
| Anti-dsDNA antibody positive | 1.79 (0.88-3.62) | 0.11 |
| UPCR > 25% decrease from  baseline to week 12 | 2.13 (1.06-4.32) | 0.03 |
| Chronicity index per unit decrease | 1.15 (1.00-1.32) | 0.05 |
| UPCR > 3 at baseline | 1.18 (0.61-2.29) | 0.62 |

UPCR=urine protein/creatinine ratio

**Supplemental Table 4: Predictors of response (complete or partial) at week 52 versus no response from logistic regression analysis for Class V cases only**

| Predictor Variable | Odds Ratio Estimate  (95% Confidence interval) | P-value |
| --- | --- | --- |
| Anti-dsDNA antibody positive | 4.33 (0.57-32.92) | 0.16 |
| UPCR > 25% decrease from baseline to week 12 | 5.86 (0.58-59.38) | 0.13 |
| Chronicity Index per unit decrease | 1.38 (0.92-2.06) | 0.12 |
| UPCR > 3 at baseline | 8.83 (0.72-108.52) | 0.09 |

UPCR=urine protein/creatinine ratio

**Supplemental Table 5: Predictors of week 52 response using multinomial regression with available data**

| Predictor Variable |  | Odds Ratio Estimate  (95% Confidence Limits) | P Value |
| --- | --- | --- | --- |
| Anti-dsDNA antibody positive | CR vs NR | 4.48 (1.01-19.86) | 0.048 |
| Anti-dsDNA antibody positive | PR vs NR | 1.74 (0.55-5.55) | 0.35 |
| UPCR > 25% decrease from baseline to week 12 | CR vs NR | 5.8 (1.66-20.61) | 0.006 |
| UPCR > 25% decrease from baseline to week 12 | PR vs NR | 1.25 (0.43-3.63) | 0.68 |
| Chronicity index per unit decrease | CR vs NR | 1.42 (1.11-1.82) | 0.0049 |
| Chronicity index per unit decrease | PR vs NR | 1.26 (1.01-1.58) | 0.041 |
| UPCR > 3 at baseline | CR vs NR | 2.20 (0.64-7.58) | 0.22 |
| UPCR > 3 at baseline | PR vs NR | 6.11 (1.87-19.95) | 0.003 |

*CR=complete response, PR=partial response, NR=No Response

**
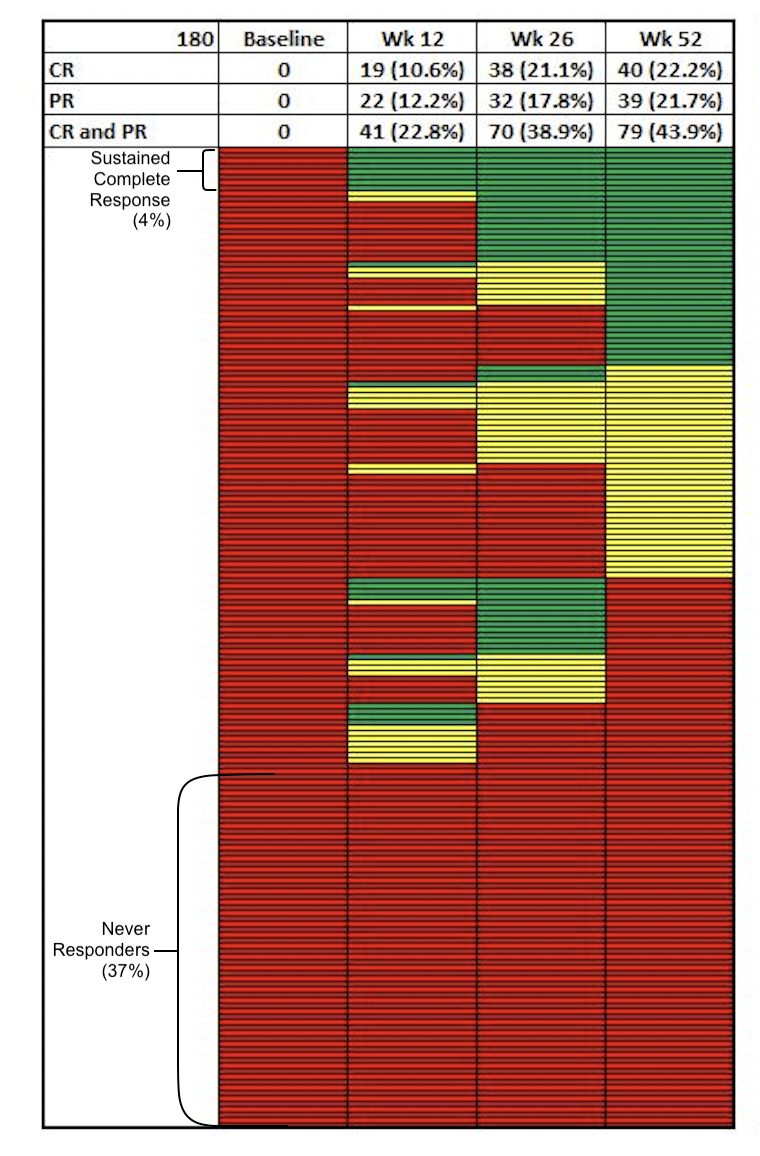
**

**Supplemental Figure 1 legend:** Temporal patterns in the response status of patients with systemic lupus erythematosus receiving standard of care therapy employing nonresponder imputation for missing data for 180 patients included. Green indicates complete response, yellow indicates partial response and red indicates no response.
